# Disgust and Other Negative Emotions in the Relationship between Mental Contamination and Post-Traumatic Stress Disorder: A Systematic Review

**DOI:** 10.62641/aep.v53i1.1822

**Published:** 2025-01-05

**Authors:** Antonia María Jiménez-Ros, Beatriz Marques, Gracia Delgado-Pardo, Ana Teresa Martins

**Affiliations:** ^1^Psychology Research Centre (CIP/UAL), Polo da Universidade do Algarve, Universidade Autónoma de Lisboa, 1150-293 Lisboa, Portugal; ^2^Centro Universitário Investigação em Psicologia (CUIP) Universidade do Algarve, 8005-139 Faro, Portugal; ^3^Departamento de Psicologia e Ciências da Educação, Faculdade de Ciências Humanas e Sociais, Universidade do Algarve, 8005-139 Faro, Portugal; ^4^Departamento de Personalidad, Evaluación y Tratamiento Psicológicos, Facultad de Psicología, Universidad de Sevilla, 41018 Sevilla, Spain

**Keywords:** mental contamination, posttraumatic stress disorder, disgust, trauma, sexual trauma

## Abstract

**Background:**

Mental contamination (MC) refers to feelings of internal filthiness associated with contamination obsessions. Ego-dystonic memories and thoughts can trigger MC, although it can also be activated by trauma, which is associated with the onset of post-traumatic stress disorder (PTSD). Research shows that MC, negative emotions and PTSD can occur simultaneously. Despite considerable interest from researchers and clinicians, to the best of our knowledge, no systematic review has been carried out on the relationship between disgust and other negative emotions with MC and PTSD. Therefore, we conducted this systematic review to summarise and synthesise the current understanding of these constructs in PTSD. The main objective of this study was to review the association between MC, post-traumatic stress disorder and trauma; the role of disgust and other negative emotions in these associations; and whether the relationship between MC and trauma is limited to traumatic sexual experiences.

**Method:**

We searched PsycINFO, Psychology and Behavioural Sciences Collection, Scopus, Web of Science, Medline, PubMed, ProQuest Dissertations and Theses, Cochrane Library, APA PsycNet, and the Online Library of the University of Algarve. We also included grey literature published in Google Scholar. The Mixed Methods Appraisal Tool was used to assess the methodological quality of the included studies.

**Results:**

Twenty studies met the inclusion criteria for the review. Among these, six studies used a randomised methodology, nine used a non-randomised methodology, and five used a descriptive methodology. The results showed that MC is associated with all types of interpersonal trauma, although the relationship with sexual trauma was the strongest. Both basic emotion and disgust sensitivity appear to be significantly associated with MC and the severity of PTSD symptoms. Similarly, other negative emotions are positively associated with MC, although the experience and reappraisal of negative emotions may potentially attenuate the experience of MC.

**Conclusions:**

Based on the findings, MC, disgust and negative emotions are important clinical constructs associated with trauma and PTSD. These results may contribute to our understanding and treatment of PTSD.

## Introduction

Fear of contamination may arise from mental contamination (MC), which is the persistent experience of feeling dirty or contaminated without physical contact with any tangible pollutant [1]. Unlike physical contamination, MC can be caused by thoughts, memories, or mental images (Rachman, 1994) [1].

Rachman (1994) [1] originally proposed MC to explain contamination-related manifestations of obsessive-compulsive disorder (OCD). However, subsequent studies have demonstrated a strong relationship between MC and post-traumatic stress disorder (PTSD), particularly in victims of sexual trauma (e.g., Tipsword *et al*. 2024 [2]) and MC has been consistently linked to more severe PTSD and negative mental health outcomes [3, 4].

Trauma, particularly sexual trauma, seems to play a significant role in the association between MC and PTSD, as suggested by Tipsword *et al*. 2024 [2]. Furthermore, Badour *et al*. [5] found that the type of assault affected the relationship between the severity of PTSD symptoms, feelings of dirtiness, and washing urges. Memories of sexual trauma have also been reported to trigger these feelings in laboratory settings [6, 7]. These findings highlight the clinical importance of better understanding MC in the context of trauma and PTSD.

However, not all studies suggest that sexual trauma is the only factor connecting MC and PTSD. Moral trauma, such as betrayal by someone with questionable moral qualities, can also induce feelings of MC [8]. Additionally, exposure to images of acts of betrayal toward others has been shown to trigger mental contamination [9]. This suggests that MC can arise from both sexual and moral trauma.

Moreover, MC is associated with specific negative emotions, such as disgust, guilt, shame, and anxiety [10]. Disgust evolved to protect organisms from physical contaminants but has been increasingly recognised as a key factor in psychological disorders involving MC [11]. Although related, disgust and MC are distinct; disgust is a basic emotion with a clear physiological response, while contamination is an evaluative process that may arise from experiencing disgust [12].

Some studies also indicate that individuals who experience MC often report high levels of disgust, both as an emotional response and as a general trait [13, 14]. This trait is associated with both the propensity for and sensitivity to disgust: propensity refers to the tendency to experience disgust in various situations, while sensitivity reflects the intensity of the emotional and physiological response [15]. Both factors seem to play a significant role in MC, and in the context of PTSD, they influence symptom severity [16].

In summary, trauma and negative emotions, particularly disgust, appear to contribute to the development of mental contamination, which, in turn, exacerbates PTSD symptoms. Recent studies suggest that sexual trauma plays a prominent role in generating MC, although other types of traumas may also be involved (e.g., Nielsen *et al*. 2024 [17]). Disgust, on the other hand, plays a central role in the experience of MC (e.g., Krause *et al*. 2022 [18]), while the role of other negative emotions in the relationship between trauma, MC, and PTSD severity remains unclear. Furthermore, to date, no literature review has considered the type of trauma and the specific role of disgust and negative emotions.

The present review aimed to attain two main objectives:

1. Analyse the research on the role of disgust and other negative emotions in the relationship between MC and PTSD, in the context of trauma.

2. Evaluate whether MC is limited to experiences of sexual trauma.

## Method

This review was carried out following the guidelines of the 2020 PRISMA declaration (**Supplementary File 1**) for systematic reviews [19].

### Eligibility Criteria

To establish the research question, the article selection, and the inclusion and exclusion criteria, the SPIDER tool was used, based on its suitability for the synthesis of qualitative and mixed-method article search [20]. This methodology allowed formulating the following research questions in a structured manner:

*Participants*: people over 18 years of age, from either clinical or non-clinical populations, who had been exposed to trauma (or induced to an experience of trauma).

*PI* (*phenomenon of interest*): the analysis of MC, considered according to the definition of Rachman [1].

*Design* (*research plan*): any exploratory, observational or experimental study design.

*Evaluation* (*main result measured*): from interviews/questionnaires or evaluation scales about the rate and severity of MC, and its relationship with other related symptoms, such as sensitivity to and propensity for disgust, PTSD symptoms, and OCD.

*Results* (*type of research*): quantitative, qualitative or mixed methods.

The inclusion and exclusion criteria are summarised in Table 1 (Ref. [1]).

**Table 1.** **Inclusion and exclusion criteria**.

Inclusion criteria	Exclusion criteria
People over 18 years of age, from clinical or non-clinical populations.	Studies with participants under 18 years of age.
The participants had been exposed to trauma or were induced to an experience or memory of trauma.	Studies in which, despite referring to the topic of interest of this review, the participants had not been exposed to an experience of trauma.
Studies that analysed MC, considered according to the definition of Rachman (1994) [1].	Articles that do not refer to the study of MC.
Articles that refer to primary sources/studies with any type of exploratory, observational and/or experimental design.	Systematic reviews, meta-analyses and any other secondary source of studies.
Quantitative, qualitative and mixed-method studies.	Non-original studies.
Studies in English or Portuguese.	Studies published in languages other than those considered in the inclusion criteria.

MC, mental contamination.

### Search Strategy

The search for the selection of studies was conducted in the PsycInfo, Psychology and Behavioural Sciences Collection, Scopus, Web of Science, Medline, PubMed, ProQuest Dissertations and Theses scientific databases, as well as in Cochrane Library, APA PsycNet and the online library of the University of Algarve. Grey literature from Google Scholar was also included. For the search, no time interval was established, and it ended in May 2023. The following keywords and Boolean operators were used, for all databases: “Mental contamination” AND Trauma* OR “Mental pollution” AND Trauma* OR “Mental contamination” AND Betray* OR “Mental pollution” AND Betrayl*.

To conduct the online search in the University of Algarve library, the keywords and Boolean operators used were: “contaminação mental” AND trauma* OR “poluição mental” AND trauma* OR “contaminação mental” AND trai* OR “poluição mental” AND trai*.

The criteria for excluding studies were: studies unrelated to mental contamination (MC), PTSD, disgust, or trauma; studies in books; book chapters; and studies unavailable in their full-text version. Additional reasons for inclusion or exclusion of studies are outlined in Table 1. No restrictions were applied in terms of variables related to sex, ethnicity, diagnosis of physical or mental health or use of pharmaceuticals.

### Article Selection Process

**Fig. 1.**
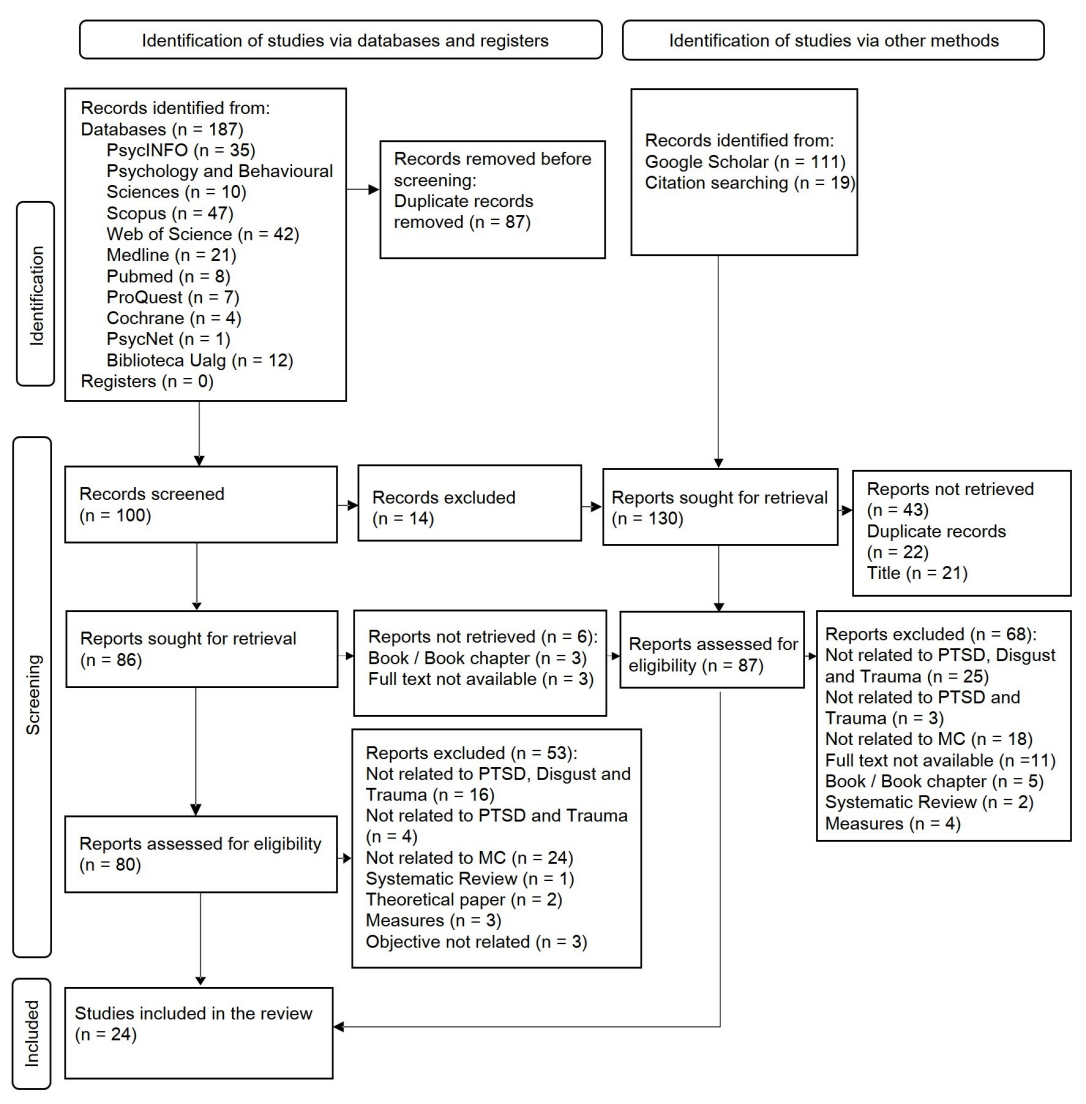
**PRISMA 2020 flow chart of searches in databases, registers and other methods**. PTSD, post-traumatic stress disorder.

The Mendeley bibliographic manager was used for the extraction of studies, which were divided into two groups: those from “databases” (N = 187) and those from “other sources” (N = 130). Duplicates in each group were removed separately and then a selection was conducted based on title and abstract screening. Subsequently, those that did not meet the inclusion criteria were removed from the sample, obtaining a total of N = 27 from databases and N = 19 from other sources. Since the selection and exclusion of studies were carried out separately for each group, according to the source of research/extraction (“databases” and “other sources”), the total sample included duplicates common to both sources, from which a total of 22 duplicate articles were discarded. Finally, the total sample was constituted by N = 24 primary studies (N = 20 articles, N = 4 dissertations) (Fig. 1).

### Risk of Bias

The quality of the included studies was evaluated through the use of the mixed methods assessment tool (MMAT). The MMAT quality tool assesses five types of studies: qualitative, quantitative randomised controlled trials, quantitative non-randomised, quantitative descriptive, and mixed-methods studies. In this review, we used the latest version of MMAT, consisting of 25 items rated on a nominal scale (Yes/No/Do not know) [21]. The tool, available online, was used to evaluate and interpret the selected articles.

Two reviewers independently conducted the review and data extraction from the included studies (BM and GDP), and then reached a consensus on the results. A third reviewer (AMJR) mediated any disagreement. The main data fields extracted included aims, country, sample, methods/design, measures, findings, and MMAT items applied to the study.

The MMAT includes two parts: a checklist with screening questions and an explanation of the criteria. Studies answering “Yes” to two screening questions proceed to detailed assessment. The final quality score is a fraction of criteria met, classified as High (80–100%), Moderate (40–60%), or Low (<20%). Only studies with a High-quality rating were included in this systematic review.

For this review, we only included studies with “high” quality (80–100% of criteria met), thus four studies were excluded: two of them did not meet the selection criteria, and the other showed “medium” quality (3/5 MMAT score).

Lastly, a total of 20 studies were included: 9 non-randomised quantitative studies, 5 descriptive studies, and 6 randomised studies (Table 2, Ref [3, 5, 12, 22, 23, 24, 25, 26, 27, 28, 29, 30, 31, 32, 33, 34, 35, 36, 37, 38]). Table 2 also presents all the measurements employed in the different studies grouped by assessment area: (a) Measurement of Mental Contamination and Related Constructs: Appraisals/Mental pollution interview; MCR: Mental Contamination Report; MPQ: Mental Pollution Questionnaire; PEMC: Post-traumatic Experience of Mental Contamination Scale; SARA: Sexual Assault and Rape Appraisals; S-CTN: Contamination Sensitive Scale; SMCS: State Mental Contamination Scale; VOCI-MC: Vancouver Obsessional Compulsive Inventory-Mental Contamination Scale; (b) Measurement of Post-traumatic Stress Disorder and Related Constructs: CAPS: Clinician-Administered PTSD Scale; PCL-5: PTSD Checklist-5-Civilian Version; PCL-C: Post-traumatic Stress Disorder Checklist-Civilian Version; IES-R: Impact of Event Scale-Revised; PTCI: Post-traumatic Cognitions Inventory; PSS-SR: Perceived Stress Scale; (c) Measurement of Disgust and Related Constructs: DPSS-R: Disgust Propensity and Sensitivity Scale-Revised; DS-R: Disgust Scale-Revised; VAS of Peritraumatic disgust and fear: visual analogue scale of Peritraumatic disgust and fear, and (d) Measurement of Trauma and Related Constructs: BSM: Betrayal Screening Measure; BSM-Self: Betrayal Screening Measure-Self; POBS: Perception of Betrayal Scale.

**Table 2.** **Characteristics of the included studies**.

Authors and year of publication	Country	Sample	Trauma experience	Study design	Study objectives	MC, PTSD and Disgust evaluation	Main results	MMAT Score
Fairbrother & Rachman, 2004 [23]	Canada	N = 50 women	Victims of sexual assault (3 months before)	Non-randomised	Explore the appearance of MC feelings after a case of sexual assault	Appraisals/Mental pollution interview	60% of the participants reported post-assault MC feelings associated with washing behaviour. The induced memory of the injury caused strong feelings of filthiness and the need for washing oneself	5/5
						SARA		
						CAPS		
						PSS-R		
Berman *et al*., 2012 [24]	USA	N = 264	History of childhood trauma	Descriptive	Evaluate relevant factors related to religion, family and trauma as predictors of MC	MPQ	It was concluded that MC is not associated with the degree of religiousness, but with the exposure to childhood trauma and maladaptive guilt-tripping strategies from the parents	5/5
		192 women						
		72 men						
Badour *et al*., 2013 [5]	USA	N = 38 women	Victims of sexual assault	Non-randomised	Examine the relationship between sensitivity to disgust, MC feelings and the severity of the symptoms of PTSD	SARA	Significant correlations were found between sensitivity to disgust and MC associated with sexual assault, with respect to the severity of the PTSD symptoms, although it was not possible to verify the permanence of these relationships in time	5/5
						CAPS		
						DPSS-R		
Badour *et al*., 2014 [12]	USA	N = 72	Victims of sexual assault before the age of 18 years (sexual victimisation)	Descriptive	Explore the relationship between disgust and MC	VOCI-MC	Peritraumatic self-centred disgust and propensity to disgust were positively correlated to MC, regardless of peritraumatic fear, post-traumatic cognitions, contamination by contact, and symptoms of psychopathology	5/5
		women				PTCI		
						CAPS		
						Peritraumatic disgust and fear		
						DPSS-R		
Adams *et al*., 2014 [25]	USA	N = 50 women	History of sexual or physical assault	Non-randomised	Evaluate the severity of the PTSD symptoms, individual differences in MC, aversion toward direct contamination (e.g., garbage), aversion toward indirect contamination (e.g., railings) and contamination OCD symptoms	VOCI-MC	Among victims of sexual assault, aversion toward contamination (direct and indirect) was positively related to MC and PTSD symptoms, but not to contamination OCD symptoms	4/5
						CAPS		
Alves, 2014 [26]	Portugal	N = 40	Perpetrators of treason	Randomised	Induce MC in perpetrators of treason, with the aim of understanding the effect on the appearance of neutralising behaviours (i.e., compulsions for washing and drinking water), feelings of disgust and negative emotions	S-CTN	The results showed that perpetrators of treason developed feelings of MC, disgust and negative emotions. These feelings are not sufficient for the development of washing compulsions	4/5
		20 women				Structured interview		
		20 men						
Ishikawa *et al*., 2015 [27]	Japan	N = 148 women	Victims of undesired sexual experience	Randomised	Investigate how undesired sexual experiences can cause MC and determine which cognitive valuation could predict MC	MCR	Remembering undesired sexual experiences may cause MC. Women who frequently recall or reflect on undesired sexual experiences are at greater risk of developing MC	4/5
						IES-R		
Jacinto, 2015 [28]	Portugal	N = 90	Victims of disloyalty	Randomised	Determine whether the memory of disloyalty may trigger negative emotions and behaviours related to MC in a group of university students	S-CTN	Remembering a situation of treason (disloyalty) triggers the appearance of negative emotions and disgust. It was not possible to demonstrate that the memory of a situation of treason generates feelings of MC or physical contamination (washing impulses) in a sample of university students	4/5
		70 women				Structured interview		
		20 men						
Pires, 2015 [29]	Portugal	N = 40	Victims of infidelity	Randomised	Experimentally induce the feeling of MC through the memory of a situation in which the participant was a victim of treason (infidelity)	S-CTN	The results showed an increase of negative emotions in the experimental group, with significant levels of disgust toward the perpetrator of treason and symptoms of MC. To a lesser degree, behaviours of washing and neutralisation were observed	4/5
		30 women 10 men				Structured interview		
Fergus & Bardeen, 2016 [30]	USA	N = 101	Victims of sexual trauma	Non-randomised	Examine the relationship between MC, tolerance to negative emotions and symptoms of PTSD	VOCI-MC	The results indicated that the difficulties to tolerate negative emotions may be a necessary condition for MC to be related to PTSD symptoms after suffering a sexual trauma	5/5
		women				PCL-5		
Brake *et al*., 2018 [31]	USA	N = 236	Victims of trauma (death threat, severe injury, rape, and accidental or violent death)	Descriptive	Analyse the relationship between MC, PTSD symptoms, mood-dependent risk behaviours, and attitudes of help search	VOCI-MC	The results indicated a positive relationship between MC and PTSD symptoms associated with different traumatic events without observing differences in terms of gender. The findings suggest that the propensity to risk behaviours is related to positive mood, although also, and especially, to negative mood and the severity of PTSD symptoms. The search for help is directly related to the severity of the PTSD symptoms, and negatively related to MC	5/5
		181 women				PCL-5		
		55 men						
Fergus *et al*., 2018 [32]	UK	N = 102 women	Victims of sexual trauma	Descriptive	Verify whether metacognitive beliefs predict greater severity of MC after suffering sexual trauma	SMCS	Metacognitive beliefs about uncontrollability and the risk of thoughts predicted greater severity of MC after an evocative task	4/5
						PCL-5		
						DPSS-R		
Ojserkis *et al*., 2018 [22]	USA	N = 250	Victims of trauma (sexual or non-sexual assault, accident/natural disaster, life threatening disease, death, etc.)	Descriptive	Investigate the association between MC, propensity and sensitivity to disgust, and OCD symptoms with respect to a traumatic experience	VOCI-MC	The results showed that MC predicted the OCD symptoms beyond the impact of sensitivity to disgust, in the victims of trauma and in those who met diagnostic criteria of PTSD. Higher levels of sensitivity to disgust strengthen the predictive association between propensity to disgust and MC	4/5
		178 women				PCL-5		
		70 men				DPSS-R		
		2 missing						
Ojserkis *et al*., 2020 [33]	USA	N = 141	Victims of interpersonal and non-interpersonal trauma	Non-randomised	Analyse possible profiles of OCD symptoms in people exposed to different types of trauma	VOCI-MC	Significant differences were found in MC between the two types of trauma (interpersonal and non-interpersonal). The results about the profiles of obsessive symptoms were more limited. In the case of interpersonal trauma, obsessive profiles with a tendency toward hoarding were found	4/5
		108 women				PCL-5		
		33 men				DS-R		
Doggett, 2020 [34]	USA	N = 42	Victims of trauma (sexual or physical interpersonal and non-interpersonal)	Randomised	Understand the indirect effect of the type of trauma on the risk of suicide mediated by MC and PTSD symptoms	PEMC	The results indicated that physical trauma, the perception of being a burden, and pain tolerance were directly related to the risk of suicide. Sexual trauma was directly related to post-traumatic MC	5/5
		25 women				PCL-5		
		17 men				THQ		
Pagdin *et al*., 2021 (Study 2) [35]	UK	N = 83	History of interpersonal treason experience	Non-randomised	Estimate the extent of the impact of the experience of interpersonal treason on people with psychological disorders	VOCI-MC	The lack of trust related to the feeling of treason causes traumatic responses that are especially associated with OCD (as well as with other symptoms, such as anxiety and depression) and MC	4/5
		69 women				IES-R		
		15 men				POBS		
Brake *et al*., 2021 [36]	USA	N = 41 women	Victims of sexual trauma with symptoms of MC	Non-randomised	Analyse the relationship between MC associated with sexual trauma and its functional connections with different negative emotions throughout the day and in a follow-up period of two weeks	PEMC	Initial and daily average MC were largely associated with higher daily average values of negative emotions. In addition to disgust, other negative emotions (shame, guilt, rage, sadness and anxiety) were correlated to sexual-trauma-related MC in the daily follow-up period of two weeks	5/5
						SMCS		
						VAS		
Tipsword *et al.*, 2022 [3]	USA	N = 41 women	Victims of sexual trauma	Non-randomised	Examine the association between the initial and daily experiences of MC and PTSD symptoms, and the mediator role of avoidance and coping	SMCS	The findings support a model of maintenance of PTSD and MC symptoms related to trauma and mediated by avoidance. No conclusive results were found with respect to approximate coping strategies	5/5
						PEMC		
						CAPS-5		
						PCL-5		
French *et al*., 2023 [37]	UK	N = 93	History of autobiographic memories as victims or perpetrators of treason	Randomised	Analyse whether OCD people obtain greater scores in MC and anxiety when faced with the induced memory of treason	VOCI-MC	The participants with OCD experienced similar increases of MC and anxiety caused by the memory of being victims or perpetrators of treason. Conversely, the participants without mental health problems showed higher MC in the case of victims of treason.	4/5
		23 men,				SMCS		
		69 women,				BSM-Self		
		1 other				BSM-Others		
Badour *et al*., 2023 [38]	USA	N = 41 women	History of sexual trauma	Non-randomised	Examine the relationships between symptoms of PTSD, OCD and MC related to sexual trauma	PEMC	The severity of OCD and MC symptoms was associated with greater severity of PTSD, especially in common symptoms such as intrusive thoughts and avoidance	5/5
						SMCS		
						CAPS-5		
						PCL-5		

Note: BSM, Betrayal Screening Measure; BSM-Self, Betrayal Screening Measure-Self; CAPS, Clinician-Administered PTSD Scale; CAPS-5, Clinician-Administered PTSD Scale for DSM-5; DS-R, Disgust Scale-Revised; DPSS-R, Disgust Propensity and Sensitivity Scale-Revised; IES-R, Impact of Event Scale-Revised; MC, mental contamination; MCR, Mental Contamination Report; MMAT, mixed methods assessment tool; MPQ, Mental Pollution Questionnaire; OCD, obsessive-compulsive disorder; PCL-5, PTSD Checklist-5-Civilian Version; PEMC, Post-traumatic Experience of Mental Contamination Scale; POBS, Perception of Betrayal Scale; PSS-R, PTSD Symptoms Scale-Self-Report; PTCI, Post-traumatic Cognitions Inventory; PTSD, post-traumatic stress disorder; SARA, Sexual Assault and Rape Appraisals; S-CTN, Contamination Sensitive Scale; SMCS, State Mental Contamination Scale; VAS, visual analogue scale; THQ, Trauma History Questionnaire; VOCI-MC, Vancouver Obsessional Compulsive Inventory-Mental Contamination Scale.

## Results

This section presents the results obtained with regard to the objectives set.

### Disgust and Other Negative Emotions in the Association between MC and PTSD in the Context of Trauma

#### Relationship of Disgust Sensitivity and Propensity with MC, and PTSD

Among the main results of the analysed studies, and considering the relationship between disgust, MC and PTSD, the study of Badour *et al*. [5] found significant correlations between sensitivity to disgust and the severity of PTSD symptoms, which would be related to the increase of MC feelings. The authors reported that the increase of MC would be associated with the persistence of PTSD symptoms (e.g., avoidance of traumatic memories) that are enhanced by negative emotions, such as disgust, guilt and shame. In turn, Badour *et al*. [12] found that, after suffering a sexual assault, self-centred sensitivity to disgust is significantly associated with MC rather than the disgust toward the aggressor. In this case, the results showed that fear and disgust toward the perpetrator were significantly related to contamination by contact, which is more strongly related to concerns about external filth.

In the same vein, Ojserkis *et al*. [22] concluded that higher scores of sensitivity to disgust would strengthen the association between MC and propensity for disgust; that is, the more negative the self-evaluation of a person about feeling disgusted, the more likely it is that the experience of disgust will lead the person to long-lasting and internalising feelings of MC and prediction of OCD symptoms. Moreover, these people may be more prone to avoiding the signs of trauma triggered by disgust, thereby also contributing to the development/persistence of PTSD symptoms.

Regarding the results about propensity for disgust and MC in relation to suffering a trauma, Ojserkis *et al*. [33] did not find any correlation for the type of trauma (interpersonal and non-interpersonal) with the presence of obsessive-compulsive symptoms mediated by MC, propensity for disgust and PTSD. The authors stated that this could be due to possible methodological errors (small samples, time elapsed since the experience of trauma, and even the conceptualisation of the types of traumas).

In contrast, Fergus and Bardeen [30] detected correlations between negative metacognitive beliefs (e.g., uncontrollability and dangerousness of one’s own thoughts) and the severity of MC after suffering sexual trauma and exposing the participants to reminders of trauma. However, they could not confirm the relationship of propensity for disgust and the severity of PTSD symptoms with MC.

#### Relationship between Negative Emotions, MC and PTSD

With regard to tolerance to negative emotions, MC and PTSD, the negative consequences and valuations of the traumatic event, along with the feeling of MC, lead the person to maintaining a state of maladaptive alert/threat and rejection toward other people [30]. In turn, while the traumatic memory persists and the feeling of internal contamination intensifies, the person may be more prone to attempting to avoid this memory, trying to find relieve from the anguish through avoidant coping strategies that facilitate the persistence and/or exacerbation of the post-traumatic symptoms and MC related to the trauma [3].

Brake *et al*. [36] analysed the evolution of MC associated with sexual trauma for two weeks. The findings showed that both initial MC and medium levels of MC were strongly correlated with different negative emotions, such as disgust, rage and anxiety, and to a lesser extent with shame, guilt, sadness and despair. On the other hand, after carrying out the daily follow-up for two weeks, the participants reported changes in the feeling of disgust and MC in an intrapersonal and individualised manner, thereby confirming the difference between these two constructs despite the superposition that could occur between them.

Three studies [26, 28, 29] experimentally induced the feeling of MC to a group of participants, from the perspective of either victims or perpetrators of treason. Two of these studies [28, 29] evaluated victims of disloyalty and infidelity, respectively; in both cases, the findings showed significant differences in the experimental group with respect to the control group. Pires [29] found significant differences in the development of feelings of filthiness related to MC, as well as feelings of disgust and greater negative affection toward the perpetrator. Jacinto [28] could not determine the existence of a relationship between disloyalty as treason and the development of MC, although this author confirmed significant differences in terms of negative emotions and the feeling of disgust. None of these two studies could confirm significant results in regard with the urge to wash and neutralisation to reduce discomfort (e.g., thinking about other topics, “drinking water”, “accepting the situation”, “focusing on the task”, “smoking”). The study by Alves [26] on perpetrators of treason confirmed the development of feelings of MC, disgust and negative emotions, although these were not sufficient to cause the urge to wash.

### Is MC Limited to Experiences of Sexual Trauma?

This systematic review analysed different types of adverse circumstances and events, as well as sexual trauma, that are related to the appearance and severity of MC and, in turn, how the person experiences it as a victim or as a perpetrator. Overall, the majority of the analysed studies focus primarily on the association between MC and sexual trauma [3, 5, 12, 23, 25, 27, 30, 31, 32, 34, 36, 38]. However, significant findings have also emerged linking MC to other types of traumas [22, 24, 26, 28, 29, 31, 33, 34, 35]. In Doggett’s study [34], a classification outlines up to eight different types of traumas accounting for both direct and indirect effects and distinguishing between interpersonal and non-interpersonal trauma [33, 34, 35]. Additionally, different results have been observed depending on whether the trauma involves the victim, as is the case in most of the studies, or the perpetrator [26, 37].

In the case of the largest subgroup related to sexual trauma, the most significant findings showed association between trauma and MC.

Specifically, three studies [23, 27, 32] showed that, for instance, the memory of a situation of sexual trauma generates MC and the urge to wash in the victims. Another three studies [5, 30, 39] found, in addition, moderate-strong associations between MC and the severity of PTSD symptoms. However, one study [32] reported that imagining a non-consented kiss was strongly associated with the experience of MC in women who had suffered a sexual trauma, although MC was not related, in this case, to PTSD symptoms.

Some studies analysed MC in the context of sexual trauma and other types of traumas but did not obtain consistent results. While three of these studies [24, 25, 34] found evidence of a significant association of MC with sexual trauma, to the detriment of other types of traumatic experiences, the rest of the studies that included participants with different types of traumas either failed to explicitly search this association or did not find it.

More specifically, the study of Berman *et al*. [24], which was carried out with students who suffered different traumatic events (emotional abuse, physical abuse, sexual abuse, emotional negligence and physical negligence), analysed variables that predict MC, as they were considered relevant for the development and increase of the severity of this construct, and consisted in certain personal experiences related to family, religion and some type of childhood trauma. The results indicated that there was no association with the degree of religiousness, but with motivational orientation toward religion and washing rituals of MC, as well as with the introduction of parental guilt and the history of childhood trauma, which predicted both washing rituals and internal contamination. The authors concluded that emotional abuse was the only type of trauma with predictive value for internal MC, whereas sexual abuse was the only trauma that predicted washing rituals.

Adams *et al*. [25] detected that fear of contamination was strongly related to PTSD symptoms in victims of sexual trauma, and that this relationship was mediated by MC only for those participants who had suffered sexual trauma (i.e., not for those with a history of physical trauma). In this sense, the participants with a background of sexual trauma [31] experienced significantly more MC and risk behaviours under negative and positive mental states than the participants with other types of traumas. Nevertheless, after adjusting for the effects of undesired sexual contact, the association between MC and PTSD symptoms remained significant.

A different study, by Ojserkis *et al*. [22], found evidence of the predictive value of MC for PTSD symptoms for the different types of traumas considered. Similarly, Ojserkis *et al*. [33] identified that MC was significantly greater in the group of interpersonal traumas (sexual or physical) compared to the group of non-interpersonal traumas (life-threatening event, disease, accident, animal assault, etc.), with no distinction between sexual and non-sexual traumas.

The results of Doggett [34] report the impact of different types of sexual, interpersonal, physical interpersonal, and non-interpersonal traumas on the risk of suicide mediated by MC and post-traumatic symptoms. Thus, physical trauma is the one directly related to suicide, whereas sexual trauma is directly related to post-traumatic MC.

Lastly, almost all the studies that explored the MC experienced by victims and perpetrators of treason obtained similar results: in Pires’ study [29], the memory of infidelity treason triggered disgust, as well as other negative emotions, internal contamination feelings, and urge to wash. In the same vein, the results of two studies [35, 37] also found an association between interpersonal treason and MC. Likewise, the memory of betrayal from the position of its perpetrator also triggered negative feelings, disgust and MC [35, 37]. The only exception was identified by Jacinto [28], who observed that the memory of betrayal was associated with negative emotions and disgust, but not with MC.

## Discussion

The aim of this review was to analyse the role of disgust and other negative emotions in the association between MC and PTSD in the context of trauma, as well as to explore the association between MC and the different types of traumas.

Fundamentally, research on MC related to disgust and other emotions, in the context of trauma, has been focused on analysing the intrusive thoughts associated with the memory and recalling of the trauma, considering the generated feeling of internal filthiness and the subsequent urge to wash. These intrusive thoughts, images and memories are central elements in the development of both PTSD and OCD, which, along the attempts to avoid stimuli that cause anxiety, are considered mediators in the appearance of MC and capable of generating a variety of different negative emotions [38, 40].

The results of the studies included in this review refer to three ways in which disgust would be related to MC. On the one hand, the studies have analysed the role of peri-traumatic disgust as a basic emotion that emerges, among others, in the context of trauma. On the other hand, sensitivity to disgust (how negatively a person assesses the experience of disgust), which predicts or precedes the manifestation of MC (thus feeling disgusted would favour the appearance of MC symptoms), would be associated with its persistence and with post-traumatic symptoms, since sensitivity to disgust causes the person to avoid the signs of the trauma that generate disgust, and would even favour the appearance of washing rituals. On its part, propensity for disgust (how easily a person feels disgusted) could be related to the persistence of OCD symptoms after recalling feelings of disgust as a response to external or internal stimuli that would even lead to the urge to wash [22]. This is a recent area of research, and while the association between disgust and MC is known, the cognitive process involved remains uncertain. In a recent study by Ouellet-Courtois *et al*. [41], the authors concluded that both disgust sensitivity and propensity are significant predictors of MC. However, disgust sensitivity also entails a negative self-disgust, in which the individual perceives themselves as a disgusting or repulsive person, leading to a sense of contamination. Therefore, the association between the experience of disgust and the perception of being repulsive seems to increase the fear of contamination. Similarly, this feeling of internal impurity is associated with trauma (violation or sexual assault), where, alongside the sense of internal MC, other negative emotions such as fear, disgust, guilt, and shame are triggered.

The results showed that negative emotions, including disgust, seem to enhance the relationship between MC and the persistence of PTSD symptoms. In the studies in which disgust was manipulated in a laboratory context, there were doubts about whether this effect could be maintained in natural environments. Similar results have been recently reported by Olatunji *et al*. [11], who, in a prospective study, observed that participants with a history of sexual abuse with or without PTSD symptoms experienced more feelings of disgust toward themselves throughout one week compared to those participants without a history of sexual abuse. In this sense, the study of Nester and Wisco [6], conducted in a natural environment, reported that disgust was associated with the memories of trauma and greater severity of PTSD symptoms.

Another important aspect related to disgust, especially concerning sexual trauma and the development of MC, is linked to the perception of self-centred peri-traumatic disgust. According to the conclusions of Badour *et al*. [12], self-centred disgust and propensity for disgust are more intensely related to MC than disgust focused on the aggressor or fear, and they lead to MC to a greater extent than intrusive thoughts associated with PTSD or contact contamination (OCD) and other psychopathological symptoms (depression). Fear and disgust focused on the aggressor are associated with contact contamination, which implies concern about external filthiness.

Based on the results of this systematic review, disgust sensitivity seems to be significantly associated with MC and the severity of PTSD symptoms [12, 25]. After suffering a sexual assault, self-centred sensitivity to disgust is significantly associated with MC, rather than disgust focused on the aggressor. Compared to previous studies, our findings are consistent with those of a systematic review carried out by Clarke *et al*. [42], who detected significantly higher rates of self-centred disgust in victims of childhood sexual abuse with a diagnosis of PTSD symptoms, compared to a healthy control group.

Similarly, Nester and Wisco [6] found that PTSD symptoms were associated with disgust and trauma reminders, but not with the type of trauma. They suggested that this may be due to the fact that their study did not assess MC, nor disgust propensity and sensitivity, which are strongly related to post-traumatic disgust and may explain the feelings of disgust among individuals with a history of sexual trauma.

According to the findings of Ojserkis *et al*. [22], higher levels of sensitivity to disgust would strengthen the association between propensity for disgust and MC. People with high sensitivity to disgust may have a greater tendency to avoid signs of trauma that cause disgust, thereby favouring the appearance and development of PTSD symptoms. In fact, in order to regulate the intense anguish related to MC, the person develops washing behaviours related to OCD symptoms. These findings are consistent with those of cognitive psychology [43], which, through tasks with eye tracking, have demonstrated that people with high propensity for disgust seem to present a bias of attentional avoidance toward disgust-generating stimuli, as well as hypervigilance of faces with expressions of disgust.

Neuroimaging studies, particularly those using functional Magnetic Resonance Imaging (fMRI), e.g., [44], could further clarify the brain regions involved in processing disgust and their interaction with areas linked to mental contamination. One key region involved in both processes is the insula, which plays a central role in interoception and emotional processing [45]. The insula is consistently activated in response to physical and moral disgust and has also shown its involvement in the experience of mental contamination [46]. This suggests that the insula may act as a shared neural pathway that modulates the emotional and cognitive aspects of both disgust and contamination. Regarding negative emotions, Fergus and Bardeen [30] stated that their study is among the first to explore the association between tolerance to negative emotions and PTSD symptoms [47] in victims of sexual trauma. In this respect, they concluded that the negative valuation of the person about the traumatic event and its repercussions cause a feeling of threat and, consequently, an avoidant and maladaptive coping reaction, in an attempt to alleviate the anguish by inhibiting the fear response with the memory of the trauma. The experience of sexual trauma would be associated with intrusive thoughts, and MC would be related to the difficulty to tolerate negative emotions, as well as with post-traumatic symptoms. Therefore, those people who link the feeling of disgust to MC more intensely will also present more severe symptoms of PTSD, thereby enhancing the appearance of other negative emotions (disgust, guilt, shame), which will reinforce the need to avoid remembering the traumatic event.

In the studies reviewed in this systematic analysis, Tipsword *et al*. [3] were among the first to explore the association between coping mechanisms, mental contamination, and trauma. Their findings suggest that individuals with more severe PTSD or MC symptoms may not exhibit a clear preference for approach coping strategies over time. However, in daily assessments, those with more intense PTSD symptoms were found to use both approach and avoidant coping strategies within the subsequent 8 to 16 hours. Current evidence tends to support the notion that avoidant coping provides these individuals with immediate relief from distress. In contrast, approach coping strategies have been shown to predict improvement in less severe PTSD and MC symptoms when assessed over a longer period of time. This highlights the importance of future research incorporating more frequent assessments over time, with a focus on a broader range of coping strategies.

Building on this line of inquiry, more recent work by Badour *et al*. [38] examined 41 women with a history of sexual trauma, conducting twice-daily assessments over 14 days to investigate the coping strategies they employed—both avoidant and approach-oriented—and their relationship with changes in mental contamination. Their findings revealed that women with more severe MC symptoms reported a more frequent use of a variety of coping strategies, including avoidant behaviours such as distraction, denial, giving up, self-blame, thought suppression, and washing behaviours, as well as approach strategies like emotional processing (identifying and understanding emotions) and emotional expression (openly sharing emotions). These findings suggest that focusing solely on the link between MC and washing behaviour may not fully capture the range of coping efforts individuals use to manage MC. While avoidant strategies may offer short-term relief, over time, individuals may attempt to alleviate distress either independently or with the support of others, underscoring the importance of emotional processing and expression.

According to Fergus and Bardeen [30], the importance of improving the tolerance to negative emotions has implications for the therapeutic intervention, regarding exposure therapy for PTSD, especially if the person has experienced sexual trauma; for adults who have suffered child abuse, it is recommended to apply a complement of cognitive therapy along with the treatment based on exposure.

Similarly, the study conducted by Brake *et al*. [36], which monitored the association between mental contamination and trauma, as well as its functional relationships with different negative emotions twice a day for a week, showed that MC and negative emotions are positively associated. However, within-subject analysis of the experience of both MC and negative emotions throughout the day revealed that the experience of negative emotions (such as disgust, shame, anxiety, anger, guilt, sadness, and hopelessness) could attenuate the experience of MC and vice versa. These results suggest that adequately reprocessing negative emotions at a certain moment of the day could lead to a later reduction in MC. Overall, these findings underscore the importance of adapting therapeutic interventions to specifically address negative emotions alongside MC to improve PTSD treatments [5]. This review also allowed observing that those studies which analysed MC in samples of victims of sexual trauma are more numerous than those which analysed this phenomenon in other types of traumas. In all the studies that evaluated samples of participants with a history of sexual trauma, MC seemed to emerge, consistently, and was associated with the severity of psychopathological symptoms [2, 5, 23, 25, 27, 30, 31, 32, 36, 37, 38], whereas the results of the studies that explored mixed types of traumas were less consistent [22, 33, 34].

Our findings are in line with those provided by Ojalehto and Abramowitz [7], who found that most of the studies were focused on the study of MC associated with sexual trauma and that, although MC also appeared as a result of other types of traumas, the strongest relationship appeared between MC and sexual trauma.

Nevertheless, the studies included in the present review that refer to interpersonal trauma, but not to sexual trauma, identified the presence of MC, almost unanimously, in both victims and perpetrators of interpersonal aggression. These results do not seem to be consistent with those of previous studies; for instance, Millar *et al*. [48] proposed that, in those studies in which the Dirty Kiss paradigm was used, the feelings of MC were triggered by imagined physical contact rather than by imagined treason. Specifically, these authors pointed out the exchange of fluids as a mechanism that triggers MC. Although we cannot assert that all types of sexual traumas involve the exchange of bodily fluids, our results do not seem to support the conclusions of this study and provide evidence in favour of MC being triggered by traumatic experiences that involve other people. MC may be experienced in these situations both as a victim and as a perpetrator of the traumatic event, and even in the absence of traumatic situations, when the morality of the individual is manipulated, as stated by Krause and Radomsky [49], where, after manipulating the moral aspect, the participants who described a past situation in which they behaved immorally presented higher levels of MC compared to those who described situations in which they behaved morally or neutral situations.

In line with Brake *et al*. [31], our results also highlight the association between MC and the severity of psychopathology, and they underline that future studies should explore the mechanisms through which MC emerges (e.g., the interpretation of the traumatic event, the moral aspects involved [8], the motivational orientation toward religion and the induction of parental guilt [24]) and how they contribute to the development and maintenance of psychopathological disorders.

Analysing the similarities and differences among them can significantly enhance our understanding of the variability in the results, with a key aspect being the type of trauma experienced by participants. While many studies focus on sexual assault, others explore different forms of trauma, such as betrayal and domestic violence. This diversity likely contributes to variations in MC and PTSD levels, as different traumas elicit distinct emotional responses that can shape individuals’ experiences.

The assessment instruments used across studies also play a significant role in shaping findings. Some studies employed scales (self-reports) specifically designed for mental contamination [12, 22, 24, 25, 31, 32, 33, 34], while others [23, 28] relied on structured interviews. These methodological differences can lead to divergent results and interpretations.

Sample characteristics, including gender and trauma history, further influence the outcomes. Studies that include both men and women often report different results regarding contamination aversion and PTSD symptoms. This variation highlights the importance of considering cultural and social contexts when analysing these relationships.

Moreover, the relationships between MC, PTSD, and other variables can be intricate. While several studies report significant correlations between MC and PTSD symptoms [3, 25, 30, 31, 38, 39], the strength and nature of these relationships vary depending on the measurement methods and contexts used. Coping strategies (e.g., Tipsword *et al*. [3]) and metacognitive beliefs (e.g., Fergus *et al*. [32]) emerge as critical factors that can mediate these connections.

Finally, the typology of the design of studies (descriptive, randomised and non-randomised) adds another layer of complexity. The variations in study typology may contribute to the discrepancies observed in findings related to MC and PTSD.

Overall, reflecting on these factors deepens our understanding of the nuanced relationships among MC, PTSD, and disgust, emphasizing the need for careful consideration of the diverse contexts in which these phenomena occur.

### Limitations and Future Directions

This review has several limitations that warrant cautious interpretation of its conclusions. Firstly, most of the reviewed studies relied on samples predominantly composed of women. Additionally, many of these studies employed convenience sampling from community participants, with financial incentives often being provided to encourage participation. These factors may restrict the generalisability of the findings.

Moreover, we must also point out that many studies relied on self-report measures, which can introduce biases such as social desirability and inaccuracies in participants’ self-perceptions, potentially skewing the results. Furthermore, recall bias may affect the accuracy of participants’ reports on traumatic experiences and emotional responses, leading to incomplete or distorted data.

Some of the studies included in the present review employed clinical analogues, while others used clinical participants, thus the obtained results may not be comparable. In some cases, the participants received a clinical diagnosis of PTSD after being recruited in the study, based merely on a semi-structured interview.

Most of the studies analysed disgust and mental contamination (MC) associated with PTSD symptoms in situations in which the person imagined the event as a victim, and very few studies explored these phenomena in relation to the role of the perpetrator of an immoral act. Although the results seem to indicate the existence of similar findings regarding MC, disgust, and PTSD symptoms between victims and perpetrators, future studies may be able to delve into the impact of these processes and the relationship between this type of event and other negative emotions, such as shame and guilt, in the perpetrator of the negative act.

Another significant limitation is the generalisability of findings, as many studies may include specific samples that do not represent diverse demographic groups—such as racial or ethnic minorities, varying socioeconomic statuses, or individuals with different sexual orientations. This limits the applicability of results to a broader range of participants and contexts, underscoring the need for research that encompasses a wider variety of demographics to enhance external validity.

Lastly, the methods for evaluating MC may have differed across the analysed studies; while some employed behavioural measures to evaluate the feeling of internal filthiness, others used self-reported instruments. Although the included studies passed quality screening processes, some had not been published or peer-reviewed when the article search was carried out.

Further research is needed along the lines of the recent study conducted by Ouellet-Courtois *et al*. [41] to explore potential moderators and mediators in the relationship between mental contamination (MC), disgust, and PTSD. Examining individual differences, such as personality traits (e.g., neuroticism or trait anxiety) and coping styles (e.g., avoidance or emotional suppression), could greatly deepen our understanding of how these emotions are experienced and managed following trauma. For instance, individuals with high levels of neuroticism may be more susceptible to intense emotional reactions and maladaptive coping strategies, potentially exacerbating feelings of contamination and worsening PTSD symptoms. Similarly, avoidance-based coping styles may hinder effective emotional processing, perpetuating the cycle between PTSD and MC.

### Clinical Implications of the Study 

Based on the research linking mental contamination to symptoms of PTSD, various clinical strategies can be derived to guide treatment and intervention for populations exposed to trauma. Firstly, the identification of mental contamination as a vulnerable factor for the escalation of PTSD symptoms suggests that therapy sessions could include the assessment and discussion of mental contamination experiences, allowing patients to recognise and tolerate the negative emotions associated with these experiences, such as disgust, shame, anxiety, and sadness [32].

According to findings from review studies (e.g., Tipsword *et al*. [3]), feelings of disgust, mental contamination, and PTSD symptoms are strongly interconnected. Avoidance coping can create a vicious cycle that intensifies these factors. For instance, attempts to avoid mental contamination may trigger more traumatic memories, while avoiding those memories can heighten feelings of disgust. In turn, avoiding disgust may further exacerbate feelings of mental contamination, perpetuating the cycle.

Recognising mental contamination as a symptom that may persist and not be alleviated by cleaning or washing behaviours may lead to the implementation of cognitive-behavioural therapy (CBT) techniques that focus on emotional processing and desensitisation regarding traumatic events [31].

Furthermore, as Brake *et al*. [36] suggest, experiencing self-disgust and mental contamination, particularly—but not exclusively—in cases of sexual trauma, can act as a barrier to seeking help. Individuals may feel responsible for the traumatic event and view themselves as unworthy of assistance. Implementing programmes to raise awareness and understanding of these concepts, along with early identification, could be valuable tools in preventing PTSD in trauma cases.

Moreover, the relationships between PTSD symptoms, risk behaviour, and negative mood states indicate the need to develop targeted interventions that help patients manage intense emotions and control impulsive or risky behaviours. Emotional regulation techniques, such as mindfulness training or coping skills, can be integral to the treatment curriculum, helping patients to move away from seeking temporary relief through harmful behaviours.

These clinical strategies are supported by findings that corroborate the complexity of the relationships between mental contamination, PTSD, and risk behaviours, as a deeper understanding of these elements can facilitate the identification of at-risk individuals and highlight the importance of prevention and treatment practices. Therefore, integrating theories and practices in PTSD treatment with a focus on negative emotions and mental contamination may result in more effective and tailored interventions to meet the needs of traumatised patients.

## Conclusions

This review aimed to understand the relationship between negative emotions, such as disgust, and mental contamination (MC) in the context of post-traumatic stress disorder (PTSD). The analysed studies allow suggesting that both disgust propensity and sensitivity are key factors in the persistence of MC and PTSD symptoms. The findings of the review should be analysed cautiously, as the studies are limited by the predominance of female samples and reliance on self-report measures, highlighting the need for more diverse and longitudinal designs.

Our findings have important clinical implications. Therapeutic interventions that enhance tolerance to negative emotions are crucial for effective PTSD treatment, particularly for victims of sexual abuse. Cognitive-behavioural therapy (CBT) techniques that focus on emotional and trauma processing are recommended. Future research should address the limitations of current studies by including more diverse populations and exploring additional factors, such as personality traits and coping styles, in order to develop more personalised and effective interventions.

## Availability of Data and Materials

Data will be available upon request to the corresponding author.

## Supplementary Material

Supplementary material associated with this article can be found, in the online version, at https://doi.org/10.62641/aep.v53i1.1822.
